# Reliability of task-specific neuronal activation assessed with functional PET, ASL and BOLD imaging

**DOI:** 10.1177/0271678X211020589

**Published:** 2021-06-02

**Authors:** Lucas Rischka, Godber M Godbersen, Verena Pichler, Paul Michenthaler, Sebastian Klug, Manfred Klöbl, Vera Ritter, Wolfgang Wadsak, Marcus Hacker, Siegfried Kasper, Rupert Lanzenberger, Andreas Hahn

**Affiliations:** 1 Department of Psychiatry and Psychotherapy, Medical University of Vienna, Vienna, Austria; 2 Department of Biomedical Imaging and Image-guided Therapy, Division of Nuclear Medicine, Medical University of Vienna, Vienna, Austria; 3 Department of Pharmaceutical Sciences, Division of Pharmaceutical Chemistry, University of Vienna, Vienna, Austria; 4 Center for Biomarker Research in Medicine (CBmed), Graz, Austria

**Keywords:** fMRI, functional PET (fPET), PET/MRI, task-specific activation, test-retest reliability

## Abstract

Mapping the neuronal response during cognitive processing is of crucial importance to gain new insights into human brain function. BOLD imaging and ASL are established MRI methods in this endeavor. Recently, the novel approach of functional PET (fPET) was introduced, enabling absolute quantification of glucose metabolism at rest and during task execution in a single measurement. Here, we report test-retest reliability of fPET in direct comparison to BOLD imaging and ASL. Twenty healthy subjects underwent two PET/MRI measurements, providing estimates of glucose metabolism, cerebral blood flow (CBF) and blood oxygenation. A cognitive task was employed with different levels of difficulty requiring visual-motor coordination. Task-specific neuronal activation was robustly detected with all three imaging approaches. The highest reliability was obtained for glucose metabolism at rest. Although this dropped during task performance it was still comparable to that of CBF. In contrast, BOLD imaging yielded high performance only for qualitative spatial overlap of task effects but not for quantitative comparison. Hence, the combined assessment of fPET and ASL offers reliable and simultaneous absolute quantification of glucose metabolism and CBF at rest and task.

## Introduction

Human brain function has been subject to research for centuries and is yet not fully understood due to its vast complexity. Thus, characterizing the neuronal response during cognitive processing is of pivotal importance and can be achieved with various approaches.

The most common imaging method to investigate task-induced changes in the brain is functional magnetic resonance imaging (fMRI) based on the blood oxygen level-dependent (BOLD) signal. Advantages are high sensitivity to changes in blood oxygenation, readily accessible MRI sequences, established tasks and high temporal and spatial resolution. However, the BOLD signal is a composite of changes in cerebral blood flow (CBF), cerebral blood volume (CBV) and blood oxygenation^1^ and consequently, only an indirect, non-specific proxy for neuronal activation. Other drawbacks are the instability of the BOLD signal for longer task durations, spurious effects such as scanner drifts^2^ and heating,^3^ field inhomogeneity,^3^ heart rate and respiratory influences,^4^ or draining veins.^5^ These are non-trivial issues and to a certain extent reasons that absolute quantification of BOLD signal changes require substantial effort.^6^

Another fMRI-based approach is arterial spin labeling (ASL) which enables quantification of CBF. Here, arterial blood is magnetically labeled to use it as endogenous contrast agent. Perfusion-weighted images are then computed from a labeled and an unlabeled image, enabling estimation of CBF.^7,8^ This absolute quantification represents a major advantage of ASL, facilitating a comparison between rest and task-specific flow changes. However, the requirement to acquire images in pairs reduces the temporal resolution and also the spatial resolution is inferior compared to BOLD imaging.^9^

Task-induced changes are also reflected in altered glucose metabolism. Hence, positron emission tomography (PET) using the glucose analogue [^18^F]FDG is a suitable tool to map neuronal activation. Indeed, task-specific changes in glucose metabolism were already assessed before BOLD imaging and ASL were introduced.^10^ A crucial drawback of this method is that measurements have to be conducted in at least two separate sessions, mostly carried out even on separate days, one at rest and another one during task execution. Changes in daily performance or resting activity between these measurements will influence the results^11^ and subjects are exposed to ionizing radiation twice. Recently, these issues were resolved with the novel approach of functional PET (fPET). The method enables the quantification of resting and task-specific glucose metabolism within a single measurement.^12,13^ The protocol was further optimized by the administration of [^18^F]FDG as bolus plus constant infusion.^14^ This increases the signal-to-noise ratio and the amount of freely available radiotracer to track even subtle task-related changes with a temporal resolution of minutes (instead of hours or days as previously required). Disadvantages include the necessity for arterial cannulation to enable absolute quantification and the radiation exposure of participants.

In sum, task-specific neuronal activation is reflected in subtle changes from the resting activity in glucose metabolism, blood flow and oxygenation. The abovementioned technical challenges and individual physiological effects might limit the detection of these changes. Hence, a valuable imaging approach is not only sensitive to task-induced changes but also has to provide high reliability during rest and task conditions. This is of pivotal importance for scientific and clinical applications to ensure that subtle effects can be robustly detected despite the variance inherent to repeated measurements. As such applications commonly aim to assess complex cognitive functions, it is essential to know the reliability for correspondingly complex tasks, since reliability of simpler tasks may not be extrapolated adequately.^15^

While ASL and BOLD imaging underwent optimization for decades already, fPET is still in its infancy and the applicability of this novel imaging approach in longitudinal studies was not yet assessed. Therefore, we conducted a test-retest study and employed a challenging task with varying levels of cognitive load, given by the video game Tetris®. The introduction of fully-integrated PET/MRI scanners enabled the simultaneous acquisition of fPET, ASL and BOLD imaging. Thus, the test-retest reliability between these imaging modalities can be most directly compared in a single scan session. We aimed to assess i) the capability of fPET to track task-specific changes with a high reliability between measurements and ii) its performance in comparison to that of the well-established modalities of ASL and BOLD imaging. For this purpose, we investigated common parameters for each modality (influx constant K_i_ and cerebral metabolic rate of glucose (CMRGlu) for fPET, CBF for ASL and parameter estimates (i.e., beta values obtained from the general linear model) for BOLD imaging) and assessed their test-retest reliability with frequently used metrics (intraclass correlation, coefficient of variation, Dice coefficient).

## Material and methods

### Participants

For this study, 53 healthy subjects were initially recruited, data from 40 were used and test-retest reliability was assessed for 20. Among the 13 drop out subjects, 7 discontinued voluntarily after the first measurement, for 1 subject arterial blood sampling failed, for 2 subjects ASL could not be acquired during the first measurement because of temporary technical challenges with the PET/MRI scanner (only affecting ASL), for 3 subjects only MRI was carried out due to arterial puncture and/or radiotracer synthesis failure. Cross-sectional data of a subsample was already included in a previous analysis.^16^ All subjects underwent a routine medical investigation at a screening visit including electrocardiography, blood tests, neurological and physiological tests, and a urine drug test. Psychiatric disorders were ruled out with the Structural Clinical Interview DSM-IV conducted by an experienced psychiatrist. Female participants additionally underwent a pregnancy test at the screening visit and before each PET/MRI measurement. Participants had to fast for at least four hours prior to the scan, including no consumption of sweetened beverages and caffeine.^17^ Exclusion criteria were weight above 100 kg for reasons of radiation protection, current or previous neurological, physiological or psychiatric disorders, current breastfeeding or pregnancy, left-handedness, substance abuse, MRI contraindications, participation in a study including ionizing radiation exposure (past 10 years) and regularly playing Tetris® or similar games (including mobile phone games) within the last 3 years. After detailed explanation of the study protocol, all subjects gave written informed consent. All subjects were insured and reimbursed for their participation. The study was approved by the Ethics Committee of the Medical University of Vienna (ethics number: 1479/2015) and all procedures were carried out in accordance with the Declaration of Helsinki. The study was registered at ClinicalTrials.gov (ID: NCT03485066).

### Study design

**Figure 1.**
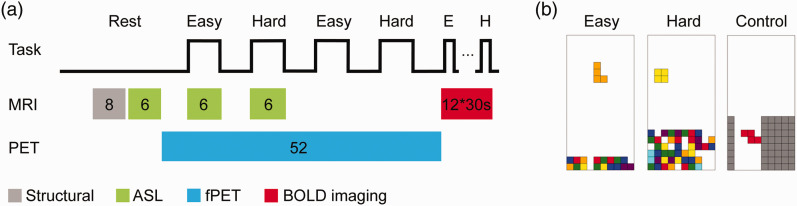
Study design and cognitive task. (a) Measurements were initiated with a structural T1-weighted image (grey, 8 mins) and ASL at rest (green, 6 mins). Thereafter, fPET was acquired (blue, 52 mins) and an adapted version of the video game Tetris® was played four times with varying cognitive load (6 mins, 2 easy, 2 hard, pseudo-randomized order) separated by resting periods. Simultaneously, ASL was acquired during one easy and one hard condition (green). BOLD data for functional connectivity was acquired during the second easy and hard task blocks, but these were not used in the current study. Immediately after fPET and ASL, BOLD data was acquired with the same task and an additional control condition (red, 12 task blocks, 30 s each, 10 s rest). (b) The task consisted of the conditions easy, hard and control, whereas the latter was only carried out during BOLD imaging. Easy and hard conditions differed by the speed of the falling bricks and the initial number of incomplete lines. In the control condition bricks had to be guided through a chimney and were automatically removed at the bottom. This figure was adapted from Hahn et al. (2020) under CC-BY license.^16^

Forty healthy subjects (20 male, mean age ± sd = 23.0 ± 3.4 years, all right-handed) underwent a single PET/MRI scan on a fully-integrated PET/MRI system (Siemens Biograph mMR, Siemens Healthineers, Germany). To assess the test-retest reliability of functional imaging parameters, a subgroup of 20 subjects (10 male, 23.1 ± 3.1 years) also underwent a second measurement (4.2 ± 0.7 weeks apart). The scans of the remaining 20 subjects (10 male, 23.0 ± 3.7 years) solely served for an independent region of interest definition. Measurements were initiated with the acquisition of a structural T1-weighted image followed by an ASL sequence during rest. Subsequently, [^18^F]FDG was administered as bolus followed by constant infusion while a complex cognitive paradigm was employed. After 8 minutes of rest, 4 task conditions were carried out with varying difficulty (6 min each, 2 easy, 2 hard, pseudo-randomized order). Each task was followed by a rest condition (5 min) where subjects were instructed to look at a black crosshair on grey background and to let their thoughts wander. Simultaneously with the task blocks, ASL was acquired during one easy and one hard condition. During the remaining task blocks, BOLD imaging was acquired for functional connectivity as described elsewhere.^16^ Immediately after fPET and ASL acquisitions, the same task was carried out in a conventional block design and BOLD data was acquired (4 easy, 4 hard and additionally, 4 control blocks, 30 s each, pseudo-randomized order), again separated by rest blocks (10 s each). Although the duration and number of task blocks differed across imaging modalities, we aimed to employ an optimal acquisition protocol for each parameter. The entire study design is depicted in Figure 1(a).

### Cognitive task

An adapted version of the video game Tetris® was carried out, representing a complex visuo-spatial motor task, which combines mental rotation, spatial planning and hand-eye coordination, thus, targeting various higher-order brain regions. The task comprised two levels of difficulty (easy and hard) to induce different cognitive loads (Figure 1(b)). The aim of the task was to complete full horizontal lines by moving and rotating bricks falling from the top of the screen. Subjects played only with their right hand on an MR-compatible button box (index/small finger: move left/right, middle finger: rotate, ring finger: move down). The two levels varied regarding the speed at which the bricks were falling and the initial number of incomplete lines at the beginning (Figure 1(b)). During BOLD acquisition, a control level was introduced where bricks had to be lead through a chimney sufficiently wide that no rotation was necessary (Figure 1(b)). For the control level, bricks were removed at the bottom. Thus, no completion of lines was possible. In general, subjects were instructed to gain as many points as possible and were explained that completion of several lines at once scores more points. Right before the start of the measurement, each condition was played once (30 s each) in the scanner to familiarize the participants with the procedure and the controls.

### PET and MRI data acquisition

The radioactive glucose analogue [^18^F]FDG was freshly synthesized every morning using FASTlab FDG cassettes with phosphate buffer formulation^18^ and a FASTlab platform (GE Healthcare). The substance was administered via a cubital vein as bolus for 1 minute followed by constant infusion for 51 minutes with an infusion pump (Syramed µSP6000, Arcomed, Switzerland, dosage: 5.1 MBq/kg, bolus speed: 816 ml/h, infusion speed: 42.8 ml/h, bolus-infusion ratio of activity: 20:80%), which was placed in an MR-shield. PET data was acquired in list-mode, enabling the retrospective definition of frame lengths during reconstruction.

A T1-weighted structural image was acquired with a magnetization prepared rapid gradient echo (MPRAGE) sequence prior to radiotracer administration (TE/TR = 4.21/2200 ms, voxel size = 1 × 1 ×1.1 mm, matrix size = 240 × 256, slices = 160, flip angle = 9°, TI = 900 ms, 7.72 min). The image was used to rule out severe brain disorders, for attenuation correction and normalization to MNI space.

A 2 D pseudo-continuous arterial spin labeling (pcASL) sequence was recorded at rest prior to radiotracer administration and during task conditions simultaneously with PET acquisition (TE/TR = 12/4060 ms, post-labeling delay = 1800 ms, labeling duration = 1.5 sec, readout duration per slice = 35 ms, voxel size = 3.44 × 3.44 × 5 mm + 1 mm gap, matrix size = 64 × 64, slices = 20, flip angle = 90°, 6 min). The labeling plane was 9 cm inferior of the center of the field of view, which was placed on the anterior-posterior commissure line. No background suppression or further optimizations were applied.

BOLD data was acquired with an echo-planar imaging (EPI) sequence following PET acquisition (TE/TR = 30/2000 ms, voxel size = 2.5 × 2.5 × 2.5 mm +0.825 mm gap, matrix size = 80 × 80, slices = 34, flip angle = 90°, 8.17 min).

### Blood sampling

Prior to each PET/MRI measurement, the individual fasting blood glucose level was measured (Glu_plasma_, triplicate measurement). Arterial blood samples were drawn from a radial artery throughout the radiotracer administration (time points: 3, 4, 5, 14, 25, 36 and 47 min) and were timed not to interfere with task performance and the MRI acquisition. Blood samples were processed as previously described.^12^ In short, whole blood activity and plasma activity after centrifugation were measured in a γ-counter (Wizard^2^, 3”; Perkin Elmer, USA). The whole blood curve was linearly interpolated and resampled to match the time points of the reconstructed PET frames. The plasma-to-whole-blood ratio was averaged across time points. The whole blood curve was then multiplied with the mean plasma-to-whole-blood ratio to obtain an arterial input function for absolute quantification.

### PET data preprocessing and quantification of glucose metabolism

**Figure 2.**
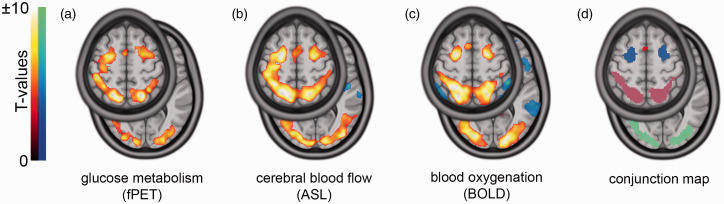
Task-specific changes and functional ROIs. Task effects were obtained with functional PET (fPET), arterial spin labeling (ASL) and blood oxygenation (BOLD). The presented maps depict group t-maps of the contrasts hard vs rest for fPET and ASL (a and b) and hard vs control for BOLD (c), all p_FWE_<0.05 corrected cluster level, height threshold of p < 0.001 uncorrected voxel level. For a robust analysis of the test-retest reliability, ROIs were determined with a conjunction analysis across all three modalities (intersection, d), revealing overlapping task changes in the frontal eye field (blue), intraparietal sulcus (pink), occipital cortex (green) and supplementary motor area (SMA, red). We focused on large, robust clusters (>500 voxels) therefore, analysis of the SMA was omitted. The slices z = 4 and z = 56 are presented in MNI space.

Data was reconstructed with an ordinary Poisson - ordered subset expectation maximization algorithm (OP-OSEM, 3 iterations, 21 subsets, matrix size: 344 × 344, slices: 127, voxel size: 2.09 × 2.09 × 2.03 mm) and binned into 104 frames of 30 s. In addition to standard corrections such as dead time and decay, attenuation and scatter correction was performed with a pseudo-CT approach^19^ based on the structural MRI acquired at the first measurement. Preprocessing and quantification steps were similar to our previous reports:^14,16,20^ SPM12 was used for head movement correction (quality = 1, registration to mean image), spatial normalization to MNI space and spatial smoothing with an 8 mm Gaussian kernel. The spatial normalization was estimated with the structural MRI. The mean PET image was then coregistered to the structural MRI and both transformations (coregistration, normalization) were applied to the dynamic PET data. Images were masked so that only grey matter voxels were present and a low pass filter with a cutoff frequency of half the task duration was applied to the time course of every voxel. A general linear model (GLM) was employed to separate task-specific and baseline metabolism including four regressors: baseline, one for each task condition (easy/hard, linear ramp function, slope = 1 kBq/frame) and the first principal component of the six movement regressors estimated during the movement correction step. The baseline term was defined with a multimodal approach (as well as an independent approach, see statistical analysis). The individual BOLD data was used to identify voxels in MNI space that exhibit significant task effects (see below and Figure 2) in the hard vs rest condition (p < 0.05 FWE corrected voxel level). These voxels were then masked out in the spatially normalized PET frames. The remaining grey matter voxels were considered inactive during the task and were averaged within each frame, yielding a task-free baseline time course. This approach has been proven useful in our previous investigation^14^ and yields similar task effects compared to a BOLD-independent baseline definition.^16^ The Gjedde-Patlak plot was applied to obtain the influx constant K_i_ with linearity set to 15 min after tracer application resulting in 3 K_i_ maps: rest, easy vs rest and hard vs rest. Finally, CMRGlu was quantified as CMRGlu = K_i_ * Glu_plasma_/LC * 100, with *LC* being the lumped constant = 0.89.

### ASL data preprocessing and cerebral blood flow quantification

The 2 D pseudo-continuous arterial spin labeling data was processed as described previously:^21^ Voxels with intensity below 80% of the mean of raw ASL data (across all voxels within each frame) were set to 0 to remove areas with insufficient signal. Movement correction was carried out with SPM12 (quality = 1) followed by calculation of the equilibrium magnetization (M_0_) map as the temporal average of all unlabeled images. The brain was extracted from the M_0_ image with the brain extraction tool implemented in FSL^22^ and the resulting mask was applied to the images of the time series. Data was spatially normalized to MNI space via the T1-weighted structural image as done for the PET data (i.e., mean image coregistered to T1, both transformations applied to all ASL data) and smoothed with an 8 mm Gaussian kernel. CBF was calculated as

(1) $$\mathrm{CBF}=\frac{\lambda \Delta MR_{1a}}{2\alpha M_{0}\{\exp\left(-\omega R_{1a}\right)-\exp[-\left(\tau +w\right)R_{1a}]\}}$$

where λ is the blood-tissue water partition coefficient (=0.9 ml/g), ΔM the difference between pairs of labeled and unlabeled images, $R_{1a}$ the longitudinal relaxation rate of blood (=0.67 sec^−1^), α the tagging efficiency (=0.8), ω the post-labeling delay time adapted for slice timing (=1800 ms at slice 1) and τ the labeling pulse duration (=1.5 sec). CBF was averaged across the time series.

Since the resulting maps acquired during task performance represent the sum of baseline and task effects, pure task-specific CBF was calculated by subtracting CBF at rest from CBF obtained during the easy and hard condition, respectively.

### BOLD-derived task changes

Data preprocessing was carried out in SPM12 as described previously:^14^ BOLD data was slice timing corrected to the middle slice, realigned to the mean image (quality = 1), spatially normalized to MNI space and smoothed (8 mm Gaussian kernel). First level analysis was performed as block design with one regressor for each task condition (control, easy, hard) in the GLM. Additionally, regressors for movement, white matter and cerebrospinal fluid (CSF) were included. The following contrasts of interest were estimated from the GLM’s beta values: control vs rest, easy vs rest, hard vs rest, easy vs control, hard vs control.

### Region of interest definition

In order to obtain an unbiased comparison between the different imaging parameters, we defined functional regions of interest (ROIs) based on all three imaging modalities similar to our previous investigation^16^ as described below. To avoid potential bias, the ROI definition and the test-retest evaluation was carried out with separate study cohorts (see study design). A group-wise one-sample t-test was performed for each modality (fPET: K_i_, hard vs rest; ASL: CBF, hard vs rest; BOLD: GLM beta values, hard vs control, all p_FWE_ < 0.05 corrected cluster level, height threshold of p < 0.001 uncorrected voxel level). The BOLD contrast hard vs control was chosen for a similar extent of task effects in the three modalities. Finally, a conjunction analysis (i.e., intersection) across the three FWE-corrected and binarized t-maps was computed to obtain task-specific ROIs. The left and corresponding right side of each ROI were merged. We focused on the largest clusters (>500 voxels) to provide a robust definition of task-relevant changes (Figure 2). These included the occipital cortex (OCC), intraparietal sulcus (IPS) and the frontal eye field (FEF) (see results), which were also identified in our previous work with a partly overlapping sample.^16^ The mean value of each ROI was extracted for further analyses.

### Statistical analysis

Statistical analyses were based on commonly reported parameters to enable comparison with previous literature, namely K_i_ and CMRGlu for fPET, CBF for ASL and parameter estimates for BOLD imaging. Similarly, frequently used metrics of test-retest reliability were used to compare imaging parameters between the two measurements. Data were visually inspected for normal distribution (Figure 4 and 5).

Quantitative comparisons were assessed using the group-wise median within-subject coefficient of variation (CoV [%] = SD/mean*100) and the intraclass correlation (ICC_3,1_, equation (2)) for each modality, each ROI and each condition:

(2) $${\mathrm{ICC}}_{3\text{,}1}=\frac{\mathrm{MSBS}-\mathrm{MSE}}{\mathrm{MSBS}+\left(k-1\right)M\mathrm{SE}}$$

where *MSBS* is the mean square between subjects and *MSE* the mean square error. MSBS and MSE were calculated from an n × k matrix with n = 20 observations and k = 2 measurements.

Spatial similarity between the task effects of the first and second measurement was assessed with the Sørensen-Dice similarity coefficient (DICE) at group level. Group level was chosen because no individual statistical maps could be computed for ASL with the current study design. Second-level analyses were performed with a one-sample t-test (n = 20) for each measurement and each task condition for all three modalities. T-maps were thresholded at p < 0.05 FWE corrected cluster level. DICE was calculated as

(3) $$\mathrm{DICE}=\frac{2|X\;\cap \;Y|}{\left|\mathrm{|X|}\right|+|Y|}$$

where *X* are all active voxels in the first measurement of one modality and one condition and *Y* are all active voxels in the corresponding second measurement.

Additional analyses were conducted to rule out spurious findings and corresponding statistics were corrected for multiple testing with the Bonferroni-Holm procedure (multiple ROIs and conditions). The use of BOLD data to identify task-specific voxels in the fPET analysis may introduce dependencies between the two modalities. Thus, we also computed task changes in glucose metabolism independent from the BOLD data by modeling the fPET baseline term with a third-order polynomial^12^ and then re-calculated reliability parameters.

Next, we investigated potential effects of task performance on test-retest reliability. Individual task performance was computed as points per minute for each condition following the original Nintendo® scoring system for Tetris®. Differences in performance between the two measurements were assessed by paired t-tests and associations with imaging parameters were calculated by Pearson’s correlation.

Finally, the effects of head motion were assessed. Average framewise displacement was computed from the realignment parameters for each subject and imaging modality.^16^ Differences in framewise displacement between the two measurements were assessed by paired t-tests and associations with coefficients of variation were calculated by Pearson’s correlation.

## Results

**Figure 3.**
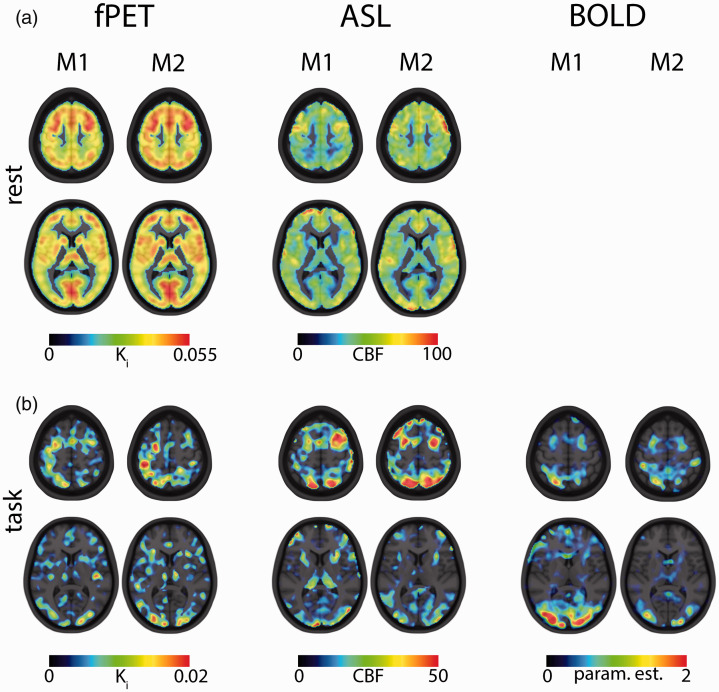
Visual comparison between measurements 1 and 2 (M1 and M2) for a representative (i.e. average test-retest reliability) subject. (a) At rest, fPET and ASL demonstrate highly similar patterns of K_i_ and CBF between the two measurements. (b) Task-specific changes in K_i_, CBF and BOLD signal show clear activations for the regions of interest frontal eye field (FEF), intraparietal sulcus (IPS) and occipital cortex (OCC) in both measurements. Similar slices as in Figure 2 are presented (z = 8 and z = 56, MNI space).

**Figure 4.**
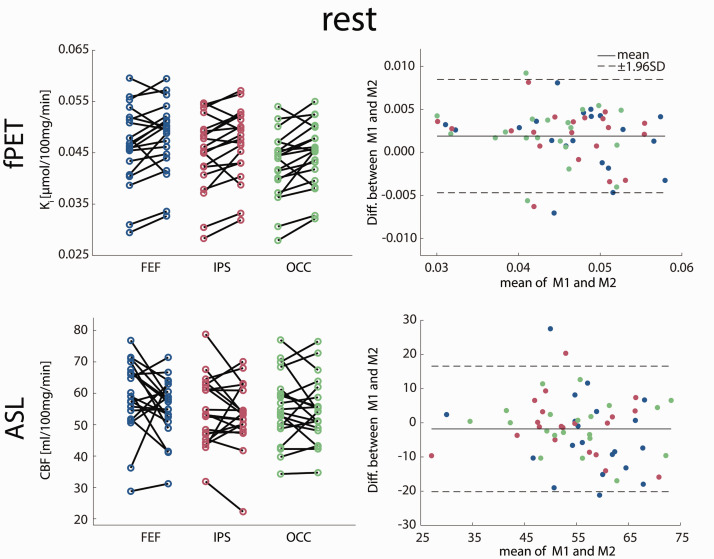
Test-retest variability at rest. Scatter and Bland-Altman plots of glucose metabolism (K_i_) and cerebral blood flow (CBF) comparing measurements 1 and 2 (M1 and M2). Both imaging modalities demonstrate good overall agreement between the two measurements with few outliers and no proportional bias. No significant differences were observed between the measurements (all p > 0.5 corrected), indicating no relevant systematic bias. See Table 1 for complementary results. Colors for ROIs match those in Figure 2. FEF: frontal eye field, IPS: intraparietal sulcus, OCC: occipital cortex.

**Figure 5.**
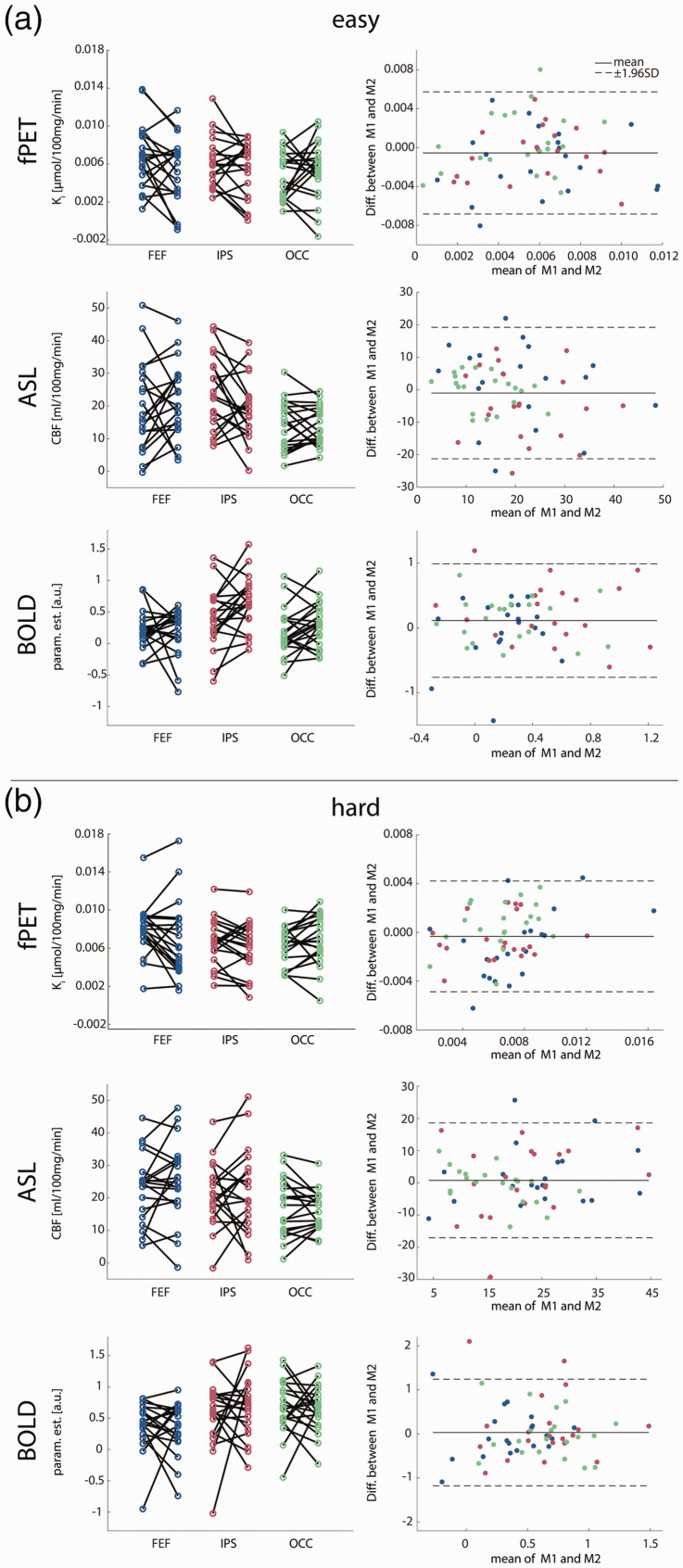
Task-specific test-retest variability. Scatter and Bland-Altman plots of glucose metabolism (K_i_), cerebral blood flow (CBF) and blood oxygen level dependent signal (BOLD, parameter estimates) comparing measurements 1 and 2 (M1 and M2). As for the rest condition, the Bland-Altman plots indicate good agreement without proportional bias or major outliers and comparable limits of agreement between the easy (a) and the hard task (b). No significant differences were observed between the measurements (all p > 0.5 corrected), indicating no relevant systematic bias. See Table 1 for complementary results. Colors for ROIs match those in Figure 2. FEF: frontal eye field, IPS: intraparietal sulcus, OCC: occipital cortex.

The following paragraphs represent the key findings. A comprehensive list of all results can be found in Table 1. Figure 3 shows voxel-wise data from a representative subject (i.e., with average coefficient of variation). Furthermore, we provide scatter and Bland-Altman plots to compare imaging parameters between the two measurements at rest (Figure 4) and during task performance (Figure 5). Reliability of CMRGlu is presented in Supplementary Table 1.

**Table 1.** Reliability of K_i_, CBF and BOLD changes.

	CoV (IQR) [%]			ICC_3,1_			DICE
	FEF	IPS	OCC	FEF	IPS	OCC	Whole brain
K_i_							
Rest	5.4 (4.2)	4.6 (2.6)	5.0 (4.0)	0.88	0.89	0.87	-
Easy vs Rest	26.1 (43.1)	27.9 (43.1)	29.9 (52.2)	0.50	0.52	0.33	0.53
Hard vs Rest	21.0 (34.8)	17.8 (16.2)	13.6 (21.3)	0.65	0.76	0.65	0.61
CBF							
Rest	10.1 (10.4)	6.4 (10.5)	5.3 (7.8)	0.39	0.69	0.76	-
Easy vs Rest	38.7 (62.0)	32.4 (28.3)	24.9 (39.3)	0.51	0.43	0.67	0.68
Hard vs Rest	15.0 (31.8)	25.1 (43.2)	17.5 (24.5)	0.67	0.48	0.72	0.73
BOLD							
Easy vs Rest	31.7 (31.2)	20.5 (37.3)	28.9 (49.8)	0.40	0.45	0.23	0.81
Hard vs Rest	16.4 (19.5)	21.1 (24.2)	24.5 (20.4)	0.41	0.13	0.09	0.78
Control vs Rest	22.3 (40.9)	38.9 (49.1)	24.5 (51.4)	0.72	0.44	0.13	0.78
Easy vs Control	61.4 (80.0)	43.4 (54.8)	40.2 (141.9)	–0.06	0.46	0.55	0.35
Hard vs Control	34.0 (63.7)	33.8 (104.4)	49.4 (51.7)	0.12	–0.08	0.12	0.70

Commonly used metrics for test-retest reliability were estimated, namely median within-subject coefficient of variation (CoV), and intraclass correlation (ICC) for each ROI and each condition. Additionally, whole-brain DICE coefficient was calculated. At resting state, K_i_ exhibited the highest reliability. During task performance, reliability dropped but was comparable between K_i_ and CBF. With BOLD imaging the best performance was achieved with the DICE coefficient, i.e. the qualitative spatial overlap of task effects between measurements.FEF: frontal eye field, IPS: intraparietal sulcus, OCC: occipital cortex.

### Regions of interest

The conjunction analysis revealed task-induced changes across all three imaging modalities in the frontal eye field (FEF), intraparietal sulcus (IPS) and the occipital cortex (OCC), depicted in Figure 2. Additionally, the precentral gyrus and supplementary motor area were identified but analyses were omitted because of the limited size of the clusters.

### Coefficient of variation

The smallest CoV were found for K_i_ at rest with a median of 4.6–5.4% for the different ROIs. Slightly higher CoV were obtained for CBF at rest, namely 5.3–10.1%. For the two task conditions (easy/hard vs rest, respectively) the CoV of K_i_ increased, ranging between 13.6 and 29.9%. Similarly, higher CoV was observed for CBF during task performance (between 15.0% and 38.7%). The CoV of BOLD changes compared to rest fluctuated between 16.4% and 38.9%. Higher CoV were obtained for the contrast of task conditions vs control (33.8–61.4%). CoV of CMRGlu was similar to K_i_ (rest: 7.0–7.9%, task: 17.4–29.3%).

### Intraclass correlation

Similar to CoV, excellent reliability was obtained for K_i_ at rest (ICC = 0.87–0.89 for the different ROIs). Contrarily, the ICC of CBF was lower during rest compared to that of K_i_ (0.39–0.76). Again, the reliability decreased during task performance, with higher ICC during the hard vs rest condition for K_i_ (0.65–0.76) and CBF (0.48–0.72). During the easy vs rest condition, ICC was lower for K_i_ (0.33–0.52), whereas ICC for CBF was similar to hard (0.43–0.67). For BOLD signal changes relative to rest, the ICC varied substantially: easy (0.23–0.45), hard (0.09–0.41) and control (0.13–0.72). Comparisons of task conditions vs control even reached negative values (−0.06–0.55). For CMRGlu, ICC was slightly lower compared to K_i_ ranging from 0.68 to 0.74 at rest and 0.31 to 0.61 during task performance.

### Sørensen-Dice coefficient

Different from the other metrics, whole-brain similarity of the task changes between the first and second measurement was highest for BOLD changes for easy vs rest (DICE = 0.81) and hard vs rest (0.78). When contrasting against the control condition, DICE slightly decreased to 0.70 for hard but dropped to 0.35 for easy. For the other imaging modalities, DICE coefficients were lower than BOLD (K_i_: easy/hard: 0.53/0.61, CMRGlu: easy/hard: 0.52/0.61 and CBF: 0.68/0.73).

### Control analyses

We have carried out a number of additional analyses to further support our findings. First, we calculated task-specific changes in glucose metabolism independent of the BOLD data. In line with our previous results this showed similar activation patterns^14,16^ and test-retest reliability (Supplementary table 2). We further investigated the potential effect of changes in task performance. Even though the subjects did not train between the two measurements there was a slight but significant increase in performance for the easy (points per minute 427 vs 710) and the hard task levels (1176 vs 1621, both p < 0.01 corrected). However, there were no significant changes in any of the corresponding imaging parameters (all p > 0.5 corrected) or any significant correlations with changes in task performance (all p > 0.5 corrected). Finally, head motion was not different between the two measurements for any of the imaging modalities (all p > 0.1 corrected). Furthermore, no association between individual framewise displacement and coefficients of variation was observed (all p > 0.5 corrected), indicating that motion did not drive the test-retest reliability.

## Discussion

In this study, the test-retest reliability of functional imaging parameters reflecting neuronal activation during a complex visuo-spatial task was assessed. The multimodal data acquisition approach facilitated a direct comparison of fPET test-retest performance to the well-established methods of ASL and BOLD imaging. We observed excellent reliability of glucose metabolism and CBF at rest. The reliability decreased during task performance for both K_i_ and CBF but was lowest for BOLD signal changes.

Measurements at resting state reflect resting activity, which plays a key role in research and clinical applications, for example, to investigate altered metabolism or blood flow in patients. A high test-retest reliability of glucose metabolism at rest was already demonstrated decades ago with 2-[1-^11^C]deoxyglucose measurements within the same day^23^ but also with [^18^F]FDG and intervals of one to twelve weeks between measurements.^24^ Similar reliability was achieved in this study with excellent ICC and small CoV. In a comprehensive evaluation of CBF, test-retest reliability was compared within session and 1 hour and 1 week apart.^25^ As expected, the CoV increased with the time between measurements, which could be based on technical challenges and varying resting activity. Although our measurements were separated by 4 weeks, we could achieve similar CoV. With the caveat of longer measurement time, K_i_ exhibited a higher reliability than CBF in terms of CoV and ICC, suggesting fPET as a robust quantitative tool to map resting activity. Other than K_i_ and CBF, estimation of BOLD changes during rest is theoretically possible by calibrating the signal^6^ but is usually not performed due to technical challenges. Consequently, a comparison to K_i_ and CBF was omitted in this work.

In addition to imaging parameters at rest, a comprehensive assessment of brain function requires analysis of changes during stimulation and performance of specific tasks. Although a drop in reliability for glucose metabolism and CBF in both task conditions was observed, the rather novel fPET technique showed similar task-specific reliability compared to the established ASL approach, however, coming at the cost of longer measurement time.

CMRGlu had slightly lower reliability than K_i_. By definition, these inconsistencies can be ascribed to changes in the LC or plasma glucose level between measurements. Since LC is assumed to be stable in healthy brains,^26^ the differences are likely to occur due to alterations in the plasma glucose levels. Thus, acquiring a temporal profile of the latter might improve the reliability. Regarding CBF, even lower ICC values were previously reported for target regions, although the measurement interval was only 1–2 days^27^ compared to 4 weeks in our study. Also robust paradigms such as finger tapping exhibited lower reliability within a measurement interval of 1 to 4 weeks.^28^ The reliability of fMRI has been subject to long-lasting discussions as results vary widely. Recently, a meta-analysis on task-specific BOLD changes, including more than 50 studies, revealed a low test-retest reliability (mean ICC = 0.40) across various task durations, test-retest intervals and task types.^15^ A similar average ICC was achieved with our task when compared to rest (ICC = 0.33), which was even lower for contrasts of task vs control (ICC = 0.17). For comparison, the average ICC of K_i_ and CBF was markedly higher with values of 0.52 and 0.58. Interestingly, the DICE coefficient (i.e., a metric that quantifies the spatial extent of overlapping activation, independent of its amplitude) was highest for BOLD changes compared to CBF and K_i_ in almost all conditions. Together, ICC and DICE indicate a high spatial overlap of task effects for BOLD, but with fluctuating effect sizes. This suggests to use BOLD more selectively, such as in research questions were simple identification of active regions is of greater importance than reliable quantitative parameters.

In summary, the test-retest reliability during task performance varied across the presented imaging approaches. A possible explanation is that each modality reflects different factors that are coupled to neuronal activation to varying degrees. While a close relationship was observed between metabolic processes and CBF, a mismatch occurs upon task performance between oxygen supply and utilization.^29,30^ During task-specific neuronal activation, the glutamatergic release is increased which triggers several neurovascular signal pathways, including vasodilating agents such as nitric oxide or prostaglandins.^31^ These factors yield a higher CBF and blood oxygen level in capillaries and arterioles. The net decrease in deoxygenated hemoglobin is the underlying mechanism of the BOLD effect.^32^ Hence, BOLD signal changes are mediated by glutamate release and influenced by the abovementioned factors. A more direct measure of metabolism is given by the radioactive glucose analogue [^18^F]FDG. Astrocytes metabolize glucose to supply neurons with energy in form of lactate.^33^ Also, in neurons themselves, glucose is transformed into ATP for action potentials and synaptic transmission.^34^ The radiotracer is irreversibly bound in cells and thus, task-specific increase in energy consumption is proportional to [^18^F]FDG uptake.^35^ Of note, also glucose metabolism influences the vasodilation by certain neurovascular signal pathways, including lactate.^36^ This complex neurophysiological interplay between the presented imaging approaches suggests to consider them as complementary and not as substitution of one another.

Another, more technical, reason for differences in test-retest variability might arise from different measurement durations between the three modalities. Acquisition times for ASL and BOLD imaging were shorter than for fPET. It is known that the temporal signal-to-noise ratio (tSNR) increases with the square root of the measurement time, which may also result in higher reliability. Hence, extending the measurement time of BOLD imaging and ASL to that of fPET (52 min) would theoretically increase the tSNR by approximately 150% and 70%, respectively (see Supplementary figure S1). Of note, this does not imply an increase in test-retest reliability by the same amount, because external influences such as physiological effects are not considered. Thus, it is likely that the test-retest reliability of task-based fMRI reaches a plateau similar to that of resting-state fMRI.^37^

Currently, BOLD imaging is often the first choice to map task changes on the whole-brain level, although several pitfalls are known. As mentioned, the BOLD effect is rather unspecific due to its signal complexity which is often compensated for by employing a control condition. However, finding a suitable control condition is challenging as it bears the risk to remove potential active regions after contrasting the conditions and has the drawback of introducing additional variance as also shown in this work.

Based on our results and aforementioned difficulties with BOLD imaging, we propose to consider fPET and ASL as a robust combined tool to tackle novel research questions using fully-integrated PET/MRI systems. This enables the simultaneous acquisition of two complementary aspects of neuronal activation. Different to BOLD imaging, fPET and ASL directly reflect glucose metabolism and CBF, respectively, making them less dependent on control conditions to achieve higher specificity. The advantage of absolute quantification further enables longitudinal comparisons during task performance and at rest. However, the acquisition requires an adaption of already established paradigms. While fPET requires longer tasks, which have to be stable for several minutes, BOLD imaging employs shorter paradigms with several repetitions. Furthermore, BOLD changes can resolve event-related task effects due to the higher temporal resolution. Differently, fPET and ASL are currently limited to block designs, although real-time and event-related designs were proposed for ASL within a specific technical setup.^38,39^ Given the current improvement of PET scanners with higher sensitivity,^40^ temporal resolutions of up to 100 milliseconds and advanced reconstruction algorithms,^41^ event-related designs also seem to be highly feasible with fPET in the near future.

In cases where the simultaneous acquisition of fPET and BOLD is favorable, we suggest short task blocks^14^ or a hierarchical design.^42^ For the latter, a fast on/off design is embedded in longer blocks, enabling a simultaneous acquisition of fPET and BOLD signal with respect to the paradigm requirements. Utilizing a dual-echo ASL sequence^43^ would further allow the acquisition of all three modalities at the same time with such a design. Still, this would imply tradeoffs mostly at the cost of the BOLD signal as ASL employs longer TR. Although a hierarchical design enables a direct comparison between the modalities, the short rest periods within a block might influence the fPET signal.

The assessment of task-specific glucose metabolism may also prove feasible in clinical routine. The current European Association of Nuclear Medicine (EANM) procedure guidelines for [^18^F]FDG PET brain imaging suggest the acquisition of a 15-30 min static image. This may also include stimulation paradigms outside the scanner to identify brain areas involved in specific task performance e.g., before surgery.^17^ However, this omits metabolic dynamics and therefore potentially misses important functional alterations connected to a disease. With fPET, resting and task-specific glucose metabolism can be robustly determined within a single 30 min measurement, enabling an optimized therapy planning (e.g., in tumor patients), but also potentially allowing for an enhanced monitoring of progression in neurodegenerative diseases.

Although high test-retest reliability of fPET was demonstrated, we have to note a few limitations. fPET currently requires arterial cannulation for absolute quantification of glucose metabolism but there is great effort to substitute the AIF with an image-derived input function which will broaden the applicability of the approach.^28,44^ Another drawback is the radiation exposure of participants (here approx. 6.4 mSv for a subject with 75 kg body weight). However, the development of PET systems with high sensitivity^40,41^ will allow further reduction of the radiation burden up to an order of magnitude.^45^ Reliability obtained with a complex visuo-spatial motor task with different task loads might be more susceptible to individual daily performance than simple tasks such as checkerboard stimulation or finger tapping. However, mapping the neuronal response to complex behavioral processes requires performance of similarly challenging tasks, which makes knowledge about the corresponding reliability an essential aspect. Another limitation is the slight improvement in task performance between the two measurements. This may be ascribed to an improved control to align the bricks, which may have resulted in altered task-specific activation in the second measurement. To diminish this effect, the measurements were separated by 4 weeks and the ICC_3,1_ was used describing consistency rather than absolute agreement. Furthermore, no changes in imaging parameters or correlations with task performance were observed. This indicates that the reported test-retest reliability was not affected by the change in task performance. Lastly, task-specific test-retest metrics were directly compared between modalities, although data acquisition varied across modalities in duration and the number of task blocks. Strictly speaking, only K_i_ and CBF were acquired simultaneously and BOLD changes immediately afterwards within the same session. These differences emerge from the different requirements of imaging modalities. fPET and ASL perform best with relatively long task blocks, whereas the BOLD signal is unstable for long continuous task durations. To meet these requirements, we employed a study design that is technically most feasible but also allows to optimize the task-specific acquisition independently for each imaging modality. Our results therefore provide test-retest data similar to commonly employed designs and it is likely that longer measurement times would increase the test-retest reliability of BOLD imaging and ASL because of a higher tSNR. However, this has to be evaluated systematically with the acquisition of more task blocks and longer measurement durations to identify a plateau of reliability.

In conclusion, matching task-specific neuronal activations were robustly detected with fPET, ASL and BOLD imaging in both task conditions. Despite its recent introduction, similar or higher reliability was achieved for fPET in comparison to the optimized and well-established modalities of ASL and BOLD imaging, albeit with a longer measurement time. Indeed, BOLD changes exhibited a substantially lower reliability, especially when using a control condition. Nevertheless, BOLD effects showed the highest qualitative overlap between the measurements, suggesting to use BOLD imaging preferably for the spatial assessment of task changes. On the other hand, the combination of fPET and ASL offers a robust tool, enabling the simultaneous absolute quantification of glucose metabolism and blood flow during rest and task performance. Considering that fPET will benefit from numerous further advancements such as progressive modeling strategies, more sensitive scanners and increased temporal resolution, an even higher reliability of this technique is to be expected in the near future. This enables the application of fPET not only in research, but also in clinical routine.

## Supplemental Material

sj-pdf-1-jcb-10.1177_0271678X211020589 - Supplemental material for Reliability of task-specific neuronal activation assessed with functional PET, ASL and BOLD imaging Click here for additional data file. Supplemental material, sj-pdf-1-jcb-10.1177_0271678X211020589 for Reliability of task-specific neuronal activation assessed with functional PET, ASL and BOLD imaging by Lucas Rischka, Godber M Godbersen, Verena Pichler, Paul Michenthaler, Sebastian Klug, Manfred Klöbl, Vera Ritter, Wolfgang Wadsak, Marcus Hacker, Siegfried Kasper, Rupert Lanzenberger and Andreas Hahn in Journal of Cerebral Blood Flow & Metabolism
