# Supplementary material for: Reliability of task-specific neuronal activation assessed with functional PET, ASL and BOLD imaging
Source: J Cereb Blood Flow Metab. 2021 Jun 2;41(11):2986–99. doi: 10.1177/0271678X211020589 (PMC8545051; doi:10.1177/0271678X211020589)
Supplement: sj-pdf-1-jcb-10.1177_0271678X211020589 - Supplemental material for Reliability of task-specific neuronal activation assessed with functional PET, ASL and BOLD imaging [file sj-pdf-1-jcb-10.1177_0271678X211020589.pdf]

SUPPLEMENTARY MATERIAL

Supplementary figure S1: *Theoretical estimation of the temporal signal-to-noise ratio (tSNR) as a function of measurement time for ASL and BOLD.*

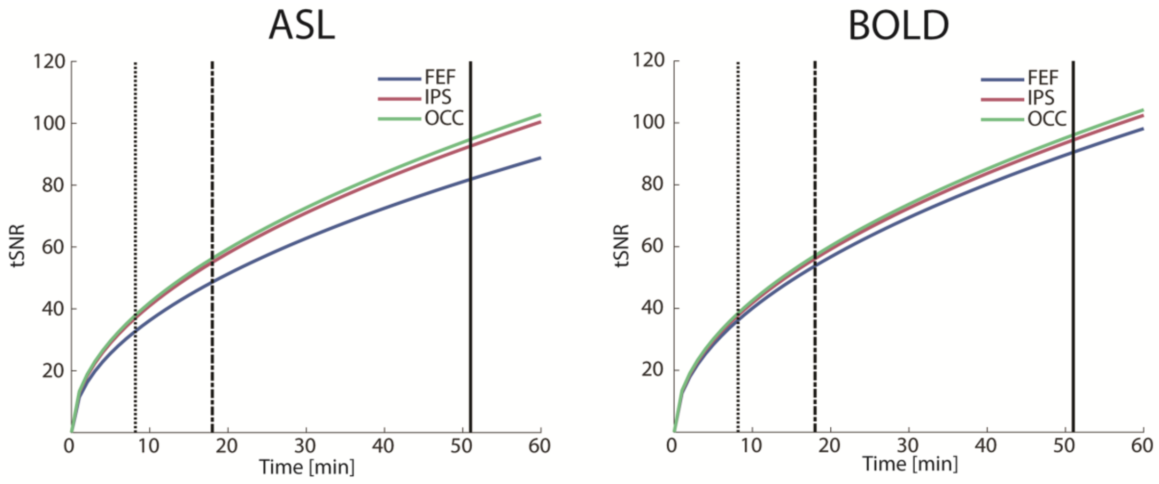

The tSNR was estimated as (x̄/sd)*sqrt(t)/sqrt($t_{modality}$) with x̄ being the mean and sd the standard deviation of the time course and t representing the measurement time (0-60 min). $t_{modality}$ represents the actual measurement time used in this study for each modality as indicated by the vertical lines (dotted: BOLD, dash-dotted: ASL, solid: fPET). Measuring ASL and BOLD imaging for the same duration as fPET (i.e. 52 min) would improve the tSNR by approximately 70% and 150%, respectively. FEF: frontal eye field, IPS: intraparietal sulcus, OCC: occipital cortex.

Supplementary Table 1: *Reliability of CMRGlu at rest and task.*

	CoV (IQR) [%]			$ICC_{3,1}$			DICE
	FEF	IPS	OCC	FEF	IPS	OCC	Whole brain
**CMRGlu**							
Rest	7.0 (11.1)	7.1 (10.0)	7.9 (8.1)	0.68	0.74	0.72	-
Easy vs Rest	29.3 (46.1)	24.4 (30.6)	27.0 (50.3)	0.44	0.53	0.31	0.52
Hard vs Rest	26.4 (30.3)	17.4 (23.3)	21.4 (16.2)	0.41	0.61	0.51	0.61

Reliability of CMRGlu was mostly comparable to K_i with the best performance at rest and a similar drop at task. Interestingly, the DICE coefficient is almost identical between K_i and CMRGlu indicating similar activation maps.

Supplementary table 2: *Reliability of K_i when calculating task-specific changes independent of BOLD data*.

	CoV (IQR) [%]			ICC$_{3,1}$			DICE
	FEF	IPS	OCC	FEF	IPS	OCC	Whole brain
**K_i**							
Rest	5.3 (3.6)	5.0 (2.3)	4.9 (3.7)	0.88	0.89	0.86	-
Easy vs Rest	23.5 (34.9)	30.0 (60.5)	43.5 (33.8)	0.64	0.58	0.32	0.52
Hard vs Rest	25.6 (16.0)	13.6 (26.8)	16.0 (32.5)	0.73	0.78	0.69	0.58

The baseline term of the fPET analysis was modeled by a third-order polynomial as described previously.[1] The approach showed similar test-retest reliability with slightly higher ICC values for the FEF but also higher variability (CoV) in the OCC.

References

1. Hahn A, Gryglewski G, Nics L, et al. Quantification of Task-Specific Glucose Metabolism with Constant Infusion of 18F-FDG. *J Nucl Med* 2016; 57: 1933–1940.
